# Molecular Dynamics Simulations of the Mutated Proton-Transferring *a*-Subunit of *E. coli* F_o_F_1_-ATP Synthase

**DOI:** 10.3390/ijms25105143

**Published:** 2024-05-09

**Authors:** Leonid A. Ivontsin, Elena V. Mashkovtseva, Yaroslav R. Nartsissov

**Affiliations:** 1 Institute of Cytochemistry and Molecular Pharmacology, 24/14 6th Radialnaya Street, Moscow 115404, Russia; yn_brg@icmph.org; 2 Biomedical Research Group, BiDiPharma GmbH, 5 Bültbek, 22962 Siek, Germany

**Keywords:** membrane proteins, F_o_F_1_-ATP synthase, mutations, proton transport, molecular dynamics

## Abstract

The membrane F_o_ factor of ATP synthase is highly sensitive to mutations in the proton half-channel leading to the functional blocking of the entire protein. To identify functionally important amino acids for the proton transport, we performed molecular dynamic simulations on the selected mutants of the membrane part of the bacterial F_o_F_1_-ATP synthase embedded in a native lipid bilayer: there were nine different mutations of *a*-subunit residues (*a*E219, *a*H245, *a*N214, *a*Q252) in the inlet half-channel. The structure proved to be stable to these mutations, although some of them (*a*H245Y and *a*Q252L) resulted in minor conformational changes. *a*H245 and *a*N214 were crucial for proton transport as they directly facilitated H^+^ transfer. The substitutions with nonpolar amino acids disrupted the transfer chain and water molecules or neighboring polar side chains could not replace them effectively. *a*E219 and *a*Q252 appeared not to be determinative for proton translocation, since an alternative pathway involving a chain of water molecules could compensate the ability of H^+^ transmembrane movement when they were substituted. Thus, mutations of conserved polar residues significantly affected hydration levels, leading to drastic changes in the occupancy and capacity of the structural water molecule clusters (W1–W3), up to their complete disappearance and consequently to the proton transfer chain disruption.

## 1. Introduction

Adenosine triphosphate (ATP) plays a crucial role in biochemical reactions. It is a macroergenic compound that facilitates various anabolic and transport processes in living organisms [1,2]. In the cell, ATP is primarily produced by the protein complex called F_o_F_1_-ATP synthase, utilizing the electrochemical gradient of hydrogen ions [3], although, some bacterial species are capable of using sodium as a coupling ion for ATP synthesis [4].

In the last decade, the development of high-resolution cryo-electron microscopy techniques has allowed researchers to obtain structural models of the enzymes of bacteria, chloroplasts, and mitochondria, enabling their examination at the atomic level [5,6,7]. ATP synthase is a membrane enzyme consisting of the two following multisubunit factors: the lipid bilayer-embedded F_o_ that carries out ion transport and the hydrophilic F_1_ that catalyzes the synthesis of ATP from ADP and P_i_ [8]. It is worth mentioning that the enzyme can also facilitate the reverse process of proton transport across the membrane, utilizing the energy derived from ATP hydrolysis [9]. The functional “core” of the F_o_ factor, present in proteins from all studied organisms, consists of a ring oligomer of 8 to 17 *c*-subunits and an *a*-subunit located on the periphery of the *c*-ring. The catalytic “core” of F_1_, that is the minimal complex capable of high-speed ATP hydrolysis, consists of three types of subunits in the α_3_β_3_γ stoichiometry [10].

Compared to the enzyme of bacteria and chloroplasts, which are monomers, mitochondrial ATP synthase, besides the catalytic “core”, contains an additional 13 subunits necessary for its dimerization and cristae formation [11]. Nevertheless, despite the differences in subunit stoichiometry and composition, the overall architecture of the complexes exhibits significant similarities across all organisms, suggesting the same for the fundamental catalytic mechanism of the enzyme [12]. Therefore, bacterial ATP synthase, representing the simplest and most extensively studied monomeric form, serves as an important model system for the investigation of the universal mechanism of enzyme function.

**Figure 1:**
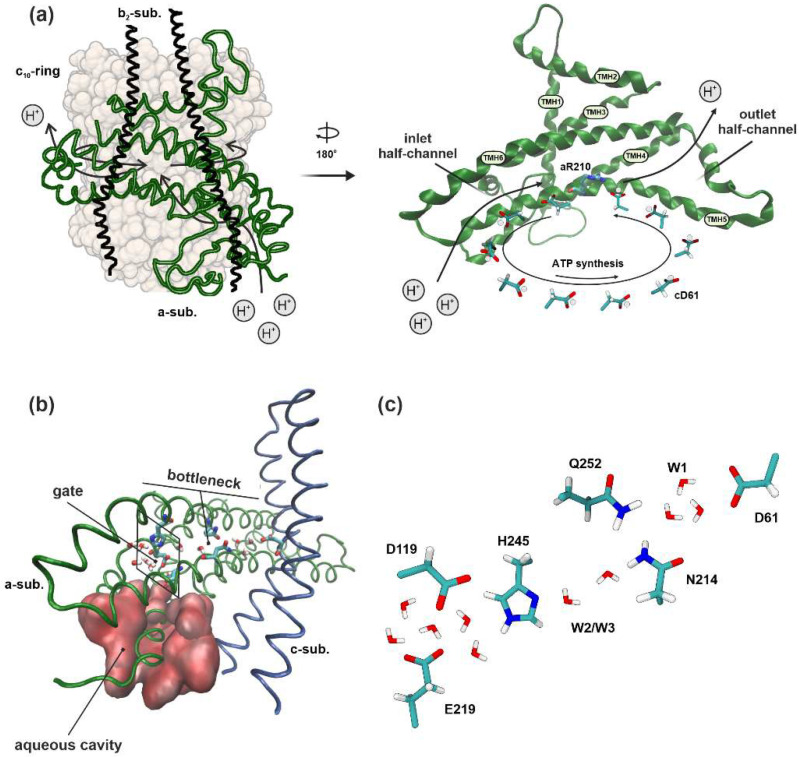
The molecular model system’s structural features. (**a**) *E. coli* F_o_F_1_-ATP synthase. On the left, the following F_o_ membrane subunits are displayed: *a*-subunit (green helices), *b*_2_-subunits (black helices), and *c*_10_-ring (gray surface). The arrows indicate the direction of proton movement during ATP synthesis. On the right, the location of proton half-channels is shown. The transmembrane helices (TMH) of the *a*-subunit and the positions of key amino acid residues (*a*R210, *c*D61) are indicated. (**b**) Detailed view of the protein inlet half-channel. Aqueous cavity is labelled as a red surface. The bottleneck is indicated, as are the amino acids side chains forming the gate. Significant polar amino acid residues (*a*E219, *a*D119, *a*H245, *a*N214, and *a*Q252) and water molecules (W1–W3) involved in proton transfer are drawn as licorice. (**c**) Scheme of the possible proton transport chain in the bottleneck of the inlet half-channel for the WT enzyme. The atoms of significant amino acids and water in half-channels possibly involved in the formation of the H-bond as located at a distance of up to 3 Å were detected. W2 and W3 clusters coordinate the proton transition between *a*H245 and *a*N214, while the W1 cluster was essential for the *c*D61 protonation.

The emergence of structural details on F_o_F_1_-ATP synthase from different organisms has led to a more comprehensive understanding of the molecular mechanisms of ATP synthesis/hydrolysis reactions [13], as well as the rotation of the *c*-subunits oligomer in the lipid bilayer [14]. This has also allowed researchers to obtain a number of structural models of proton half-channels and to identify key amino acid residues in the F_o_ factor [5,15,16]. It has been established that the transmembrane helices *a*TMH3-6 of the *a*-subunit are oriented almost horizontally in the membrane plane and are adjacent to the surface of the *c*-ring, forming an extensive interacting area concentrated around the significant residue *a*R210 in the *a*TMH4 and the key H^+^-binding residue *c*D61 in the *c*TMH2 [17]. The inlet half-channel (from the periplasmic side) passes between *a*TMH3-6 to *a*R210. The outlet half-channel (from the cytoplasmic side) is likely located at the interface between *a*TMH5-6 and *c*TMH2, extending from *c*D61 to the cytoplasmic surface (Figure 1a) [18]. The proton transfer in these two noncoaxial half-channels contributes to the protonation/deprotonation of *c*D61 residues, leading to rotor rotation and inducing conformational changes in the F_1_ catalytic factor for ATP synthesis [12]. However, the exact trajectory of proton movement is still not established, and multiple questions regarding this process remain unresolved. Moreover, the participation and the role of significant amino acid residues seems to be the point of investigation [19,20]. Thus, static cryo-EM structures have not provided clarity on the mechanism of H^+^ transport inside the channels, the impact of specific amino acid residues on this movement, and the involvement of water molecules in this process.

Since proton transport is the fastest process in the catalytic cycle of ATP synthase, it almost does not contribute to the overall enzyme rate [21]. However, the activity of the enzyme can be fully blocked if the proton transfer pathway is structurally modified. This phenomenon is due to a high sensitivity of the membrane F_o_ factor to mutations in the proton half-channel area, leading to the functional blocking of the entire protein. A series of experimental studies conducted even before the structures of F_o_ were obtained have identified amino acid residues which were functionally significant for proton transport using mutant strains of *E. coli* [22,23,24,25,26,27,28].

The efficiency of ATP synthase was considered to correlate with mutants’ growth yield in a succinate-limited medium since the protein is necessary for oxidative phosphorylation. Mutations in the *a*-subunit have been shown to affect F_o_F_1_-ATP synthase, either by disrupting the assembly of the membrane F_o_ factor due to a structural defect or by altering amino acids crucial for the proton transport process. To determine whether a certain mutation affects proton transfer, the permeability of membrane vesicles stripped of F_1_ was examined. Mutations that impact F_o_-mediated proton translocation typically reduce the ability of F_o_ to collapse proton gradients. Furthermore, ATP-induced acidification of inverted membrane vesicles was used to evaluate the ATPases capacity to carry out H^+^ transfer associated with ATP hydrolysis [25].

The results of mutational studies of the *E. coli a*-subunit amino acids *a*N214, *a*E219, *a*H245, and *a*Q252 have shown that substituting these residues with other polar and nonpolar groups led to the decrease in both ATP synthesis and passive H^+^ translocation activity, and also changed the activity of ATP hydrolysis to varying degrees [26,27,28]. Therefore, these amino acids were proposed to line the walls of the half-channels and have a significant impact on proton transport. Cryo-EM structures of the *a*-subunit have confirmed the location of these residues in the inlet half-channel [5]. While high-resolution structures along with experimental studies of mutant strains of bacterial ATP synthase have largely elucidated the mechanism of ion transport via protein, molecular dynamics (MD) simulations can complement experimental data by providing valuable insights for understanding the process of proton transport at the atomic level.

Previously, we performed molecular dynamics simulations of the membrane F_o_ factor of *E. coli* ATP synthase embedded in an aqueous environment and a native lipid bilayer containing cardiolipins (CL) [29]. This provided important insights into the proton transport pathway, particularly how and where they can pass through the F_o_ half-channels. The dynamics of significant amino acids side chains and protein hydration were investigated. Conservative localizations of structural water molecule clusters critical for proton transfer were detected. The resulting proton transport chain of polar amino acid residues and water molecules serves as the basis for an interpretation in mutagenesis studies [29]. In the present study we conducted MD simulations to perform mutational analysis for the identification of functionally important amino acids in the described chain. The effects of nine different mutations in *a*-subunit were described, wherein the importance of the residue for the proton transport process was considered by the probability of the conservation of the possible transfer pathway. The results of the mutational analysis showed that substitutions of many conservative polar amino acids led to the changes in the hydration of the inlet half-channel, which in turn affected the proton conductivity.

## 2. Results

### 2.1. Proton Transport Chain in the Inlet Half-Channel of E. coli F_o_-ATP Synthase Embedded in a Native Inner Bacterial Membrane

In the previous study, MD simulations of the F_o_ membrane part of a wild type bacterial ATP synthase embedded in a native lipid bilayer with a PE:CL ratio of 3:1 were performed (Figure 1a). Our focus was on determining the possible trajectory of proton movement through the half-channels. For this purpose, the mutual arrangement of amino acids and water molecules was investigated, as it is believed that the polar groups of the residue side chains and the oxygen atoms of water molecules can influence proton movement by forming short-lived bound states with it [30]. On the basis of the MD trajectories, we detected the atoms of significant amino acids and water in the half-channels involved in the formation of the Hbond network and located at a distance of up to 3 Å (the approximate length of a hydrogen bond). It is assumed that there is the possibility of direct proton transfer between these atoms and they could participate in the proton transfer chain, although the H^+^ transfer itself was not simulated [29].

It was found that the inlet half-channel had a composite structure, including an aqueous cavity in the protein *a*-subunit through which protons penetrated into the half-channel, and the narrow tract in the region near the key *c*D61 residue of the *c*-subunit. In this bottleneck, a sequence of conserved amino acid residues (*a*E219, *a*D119, *a*H245, *a*N214, and *a*Q252) forming a proton transfer chain was discovered (Figure 1b). A proton from the aqueous cavity could reach the significant amino acid *a*E219 and then move towards *c*D61. Additionally, we detected the localization of three structural water molecule clusters (W1–W3) that were necessary for the proton transport continuity. Cluster W1 was observed near *c*D61 and was essential for the protonation of aspartate. W2 and W3 were located between *a*H245 and *a*N214 and could coordinate proton transfer between these residues, since the distance between them rarely fell below 3 Å (Figure 1c). Stable spatial positions (SP) of the significant amino acids’ side chains of the *a*-subunit, referred to as SP1–SP3, were also identified. In particular, it was shown that the repositioning of *a*N214 in SP1 or SP3 was a necessary condition for facilitating proton transport [29].

Thus, the proton transport chain consisting of conserved amino acid residues (*a*E219, *a*D119, *a*H245, *a*N214, and *a*Q252) and water molecules (W1–W3) in the inlet half-channels of the bacterial F_o_F_1_-ATP synthase was identified. It turned out to be unstable as it required the presence of certain water molecules’ locations and the specific distances between proton binding sites. Therefore, it is assumed that mutations of these significant residues from the bottleneck may disrupt the enzyme’s function. The results of experimental mutations of the *E. coli* ATP synthase *a*-subunit showed that substitutions of some conserved polar amino acid residues led to full or partial loss of proton conduction through F_o_ [22,23,24,25,26,27,28]. These observations suggested that residues may have functionally different effects on transport, either participating in direct proton transfer or facilitating half-channel hydration. In order to identify the role of certain amino acids in the described proton transfer chain, a mutational analysis using 150 ns molecular dynamics simulations for each mutant was conducted. MD simulations seem to be a suitable theoretical method to study the transport pathway at the atomic level, as a network of amino acid residues and water molecules. Based on the wild type structure, nine different mutations were constructed in the *a*-subunit via the following substitutions: *a*E219G, *a*E219Q, *a*H245G, *a*H245S, *a*H245Y, *a*E219H/*a*H245E, *a*N214L, *a*N214H, and *a*Q252L. The mutations involved alterations in the polarity of the residues, their placement in the polypeptide chain, or the length of the side chain. Moreover, the selection of mutants was based on experimental data, as the majority of functionally important amino acids have been recognized and examined through *E. coli* mutant strains [22,23,24,25,26,27,28]. All obtained mutants were preliminary tested for the stability of the *a*-subunit structure.

### 2.2. Effect of Mutations on the Stability of the a-Subunit Structure

To evaluate the impact of mutations on the *a*-subunit stability, the change in Gibbs free energy of protein folding ΔΔG between the wild type and mutant enzyme was calculated using two programs, FoldX [31] and Eris [32], with similar algorithms using the three-dimensional structure of the protein. For almost all mutations, the calculated ΔΔG values were comparable between the two tools and were negative or close to zero, indicating a neutral or stabilizing effect. Based on this, it can be assumed that the considered mutations should not have a destructive impact on the *a*-subunit structure. Experimental substitutions of all these residues also did not disrupt the assembly of the membrane part of protein [26,27]. However, the absence of a significant influence on the overall folding stability does not guarantee that the mutations will not affect the proton transport process. In addition, the present algorithms for estimating ΔΔG still have a root mean square error close to 1 kcal/mol. This means that any explanation based on predicting the ΔΔG of mutations will have a relatively high degree of uncertainty [33].

The structures of mutant *a*-subunits were also predicted using AlphaFold to evaluate the effect of amino acid substitutions on protein folding. However, the developers of AlphaFold do not guarantee that it will generate an unfolded protein structure in the presence of a sequence containing a destabilizing point mutation [34]. Indeed, 3D folded structures of the *a*-subunits were obtained for all considered mutations. The average per-residue local distance difference test (<pLDDT>) confidence scores ranged from 0 to 100 and was used to evaluate the extent of predicted protein reproduced in the reference structure. For all predicted structures, the <pLDDT> was above 95, indicating the high accuracy of the models obtained using AlphaFold. Root mean square deviations (RMSD) were also calculated to measure the similarity between the wild type *a*-subunit structure [PDB ID: 6VWK] and mutants obtained with AlphaFold (Table 1). Lower RMSD values indicate a higher similarity between the structures. The results showed no noticeable differences and the average RMSD value for all mutants built by AlphaFold was 1.59 Å.

Based on the original structure of the wild type enzyme [PDB ID: 6VWK], molecular models with various mutations of the *a*-subunit residues were also obtained. MD simulations were used to investigate the impact of selected mutations on the conformational stability of the *a*-subunit by calculating the following parameters: root mean square deviations (RMSD), root mean square fluctuations (RMSF), radius of gyration (RG), number of hydrogen bonds (Hbond), and the solvent accessible surface area (SASA). The average values of all calculated parameters obtained from the 150 ns MD simulations are presented in Table 1.

**Figure 2:**
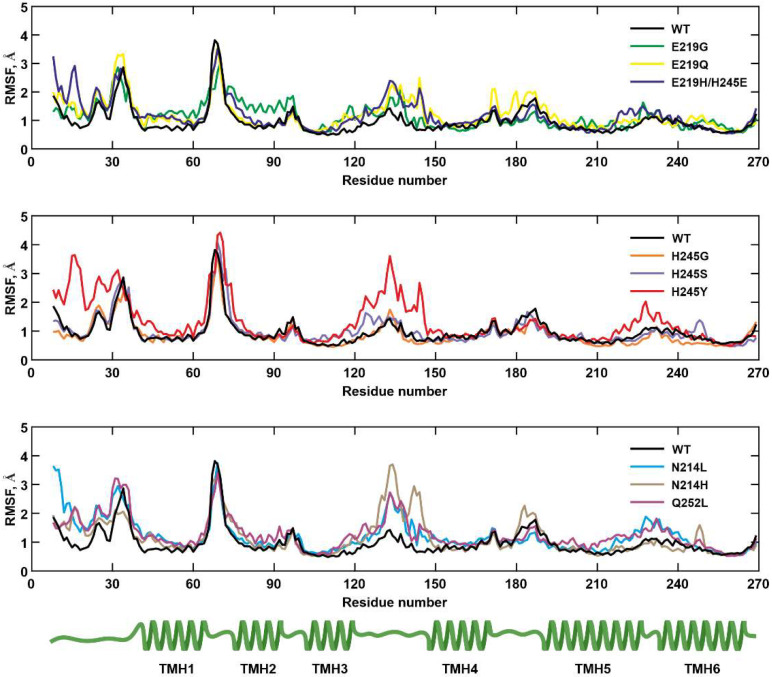
The RMSF of backbone atoms for *a*-subunit of WT and nine mutations. At the bottom is a schematic representation of the topology of the *a*-subunit that is characterized by six transmembrane helices. Hereafter, each color of the graph corresponds to the certain mutation.

The overall changes in the stability of the *a*-subunit with mutations were examined by calculating the RMSD of the backbone atoms. The average RMSD values did not exceed 3 Å, indicating that the model systems maintained their structural integrity during the MD simulation. Noticeable structural changes occurred with the *a*H245Y and *a*Q252L mutations, as the RMSD for these substitutions was 2.91 Å and 2.94 Å, respectively. Calculated RMSF values of backbone atoms from 271 amino acids in WT and mutants showed that the N-terminal part, the residues at positions 65–70 (the loop between *a*TMH1 and *a*TMH2) and at positions 121–143 (the loop between *a*TMH3 and *a*TMH4), exhibited the highest fluctuations in all systems, while the residues forming proton half-channels (*a*TMH3-6) were less mobile (Figure 2). The average RMSF value was 1.17 Å, while the highest residue mobility was observed in mutants *a*H245Y and *a*Q252L, with average RMSF values of 1.40 Å and 1.30 Å, respectively. In the case of the *a*H245Y, substitution in the region of *a*D119, *a*E219, and *a*H245Y showed an increase in the distance between *a*TMH3 and *a*TMH6 due to the displacement of *a*TMH3. The substitution of the *a*Q252 with nonpolar leucine led to an increase in the distance between *a*TMH5 and *a*TMH6 in the region of *a*N214 and *a*Q252L, as well as a shift of residues in *a*TMH5. The impact of these conformational changes on the proton transport chain is discussed below. 

The solvent accessible surface area (SASA) is considered an indicator of the protein size. All examined amino acid substitutions did not show any significant changes in SASA values compared to WT. Although mutations *a*H245Y and *a*Q252L also exhibited some of the highest SASA values (1.37 × 10^4^ Å^2^ for *a*H245Y and 1.32 × 10^4^ Å^2^ for *a*Q252L), which are likely related to conformational changes in the *a*-subunit. Radius of gyration (RG) reflects the overall compactness and size of the protein system during MD simulations. The average RG values ranged from 22.15 Å to 22.47 Å, meaning that all systems remained compact. Another parameter reflecting protein stability is the number of intramolecular hydrogen bonds (Hbond) formed between protein residues. All the considered mutations did not affect the number of Hbonds, and the average value was 60. Thus, based on the estimations of the ΔΔG values along with structural data predicted by AlphaFold and MD simulations, it was concluded that the examined mutations in general did not have a high destabilizing effect and did not lead to the *a*-subunit unfolding. However, the observed conformational changes in some mutations must be carefully considered to assess the effect on the proton transport chain.

### 2.3. The Role of Significant Amino Acid Residues in the Process of Proton Transport

Full-atom molecular dynamics simulations of the mutated bacterial ATP synthase membrane part were performed to determine the certain amino acid residue’s functional role in the proton transfer chain. We compared these obtained findings with our previous simulations of the wild type enzyme, taking into account data from site-directed mutagenesis experiments. The structural dynamics of the mutated amino acid residue side chain and those nearby, as well as protein hydration, were given special attention when analyzing the MD trajectories; the importance of the residue for the proton transport process was considered via the probability of the conservation of the possible transfer pathway.

#### 2.3.1. Mutations *a*E219

*a*E219, along with *a*D119 and *a*H245, are located at the beginning of the proton transfer chain in a narrow tract near the key *c*D61 (Figure 1b,c). In the wild type enzyme these residues were highly hydrated. Water molecules penetrated via the aqueous cavity into the inlet half-channel up to these amino acids, forming highly branched chains of hydrogen bonds through which the proton could easily reach the residues from the periplasm. The side chains of *a*E219, *a*D119, and *a*H245, which are located on three transmembrane helices of the *a*-subunit (on *a*TMH5, *a*TMH3, and *a*TMH6, respectively), overlapped the half-channel, forming something similar to a gate that hindered the passage of water molecules deeper into the half-channel towards *c*D61.

**Figure 3:**
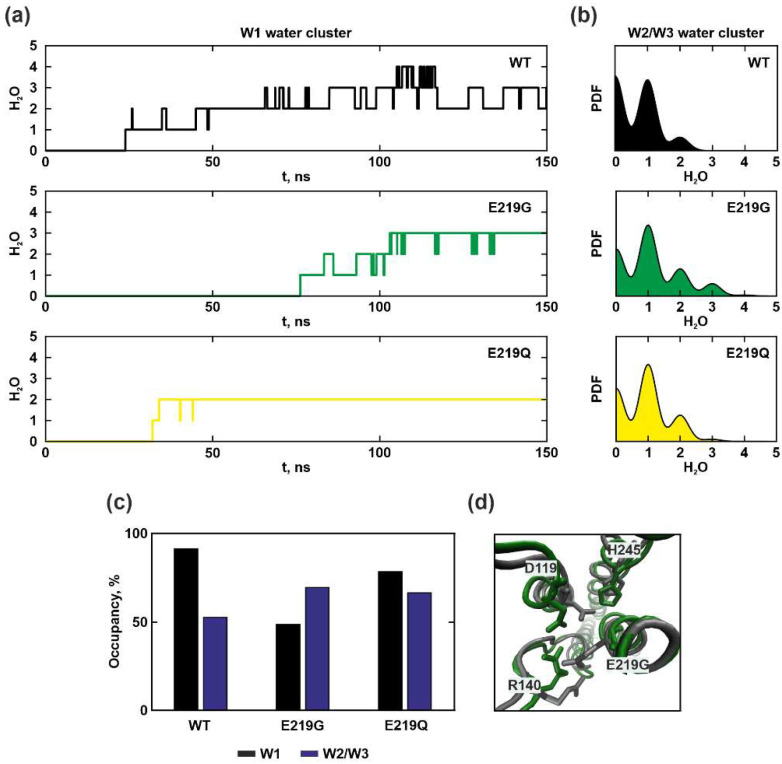
Molecular dynamics simulation results for WT, *a*E219G, and *a*E219Q. (**a**) The time propagation of the water molecules’ number in W1 cluster localization. (**b**) Probability density functions of the water molecules’ number in W2/W3 clusters localization, reflecting the capacity of the clusters. (**c**) The water occupancy of clusters (i.e., how frequently the water occurs in the localization throughout the trajectory) is shown in black for W1, blue for W2/W3. (**d**) Superimposition of the final molecular models obtained at 150 ns simulation for WT (grey) and *a*E219G (green). The change in the relative position of the *a*E219, *a*D119, *a*R140, and *a*H245 residues side chains forming the gate is demonstrated.

In the case of the *a*E219G or *a*E219Q mutations, a slowdown in the penetration of water molecules to W1 cluster localization in the depth of the inlet half-channel near *a*N214, *a*Q252, and *c*D61 was observed, although in the wild type structure these clusters filled up during the first 30 ns of simulation (Figure 3a–c). The occupancy, that was determined by calculating the percentage of the simulation time during which water molecules were observed in cluster localization, was slightly lower for W1 compared to the WT, but not for W2/W3. Water molecules were less frequently observed in the W1 cluster, leading to difficulty in *c*D61 protonation. Although these substitutions also resulted in a slight increase in capacity (number of water molecules in a cluster) of W2/W3 clusters. In both mutants, these effects may be related to the reposition of the *a*R140 residue side chain, located nearest to *a*E219, also coming into contact (at a distance of less than 3 Å) with *a*D119 for most of the simulation time (Figure 3d). Thus, in the case of the *a*E219 substitution with glycine or glutamine, the possible proton transfer pathway remained almost unchanged when compared to the wild type, however, the change in a local charge led to a hindrance in the penetration of water molecules into the depth of the inlet half-channel.

Experimental studies have shown that the *a*E219G mutant strains grew on a succinate medium via oxidative phosphorylation (i.e., maintaining ATP synthesis) and their membranes demonstrated ATP-coupled proton translocation with an efficiency of 60% to 80% of the wild type [22]. A single substitution of *a*E219Q led to the formation of a strain that grew poorly on succinate. Membrane preparations of this mutant were poorly permeable to protons and the activity of F_1_-ATPase was inhibited by approximately 50% upon binding to the membrane F_o_ factor [23]. Thus, based on experimental data along with MD simulations, it was concluded that the residue *a*E219 did influence hydration, facilitating the penetration of water molecules deeper into the inlet half-channel, but it did not directly play a crucial role in proton translocation.

#### 2.3.2. Mutations *a*H245

The residue *a*H245 was the one that transfers H^+^ from the gate to *a*N214. In the wild type, the average distance between them was 4.41 Å due to the high mobility of the *a*N214 side chain (presence of SP), and it rarely reached values below 3 Å, which hindered the proton movement in this region. However, the localization of structural water molecule clusters W2 and W3 amidst *a*H245 and *a*N214 were detected, which could facilitate the proton transfer between these residues.

**Figure 4:**
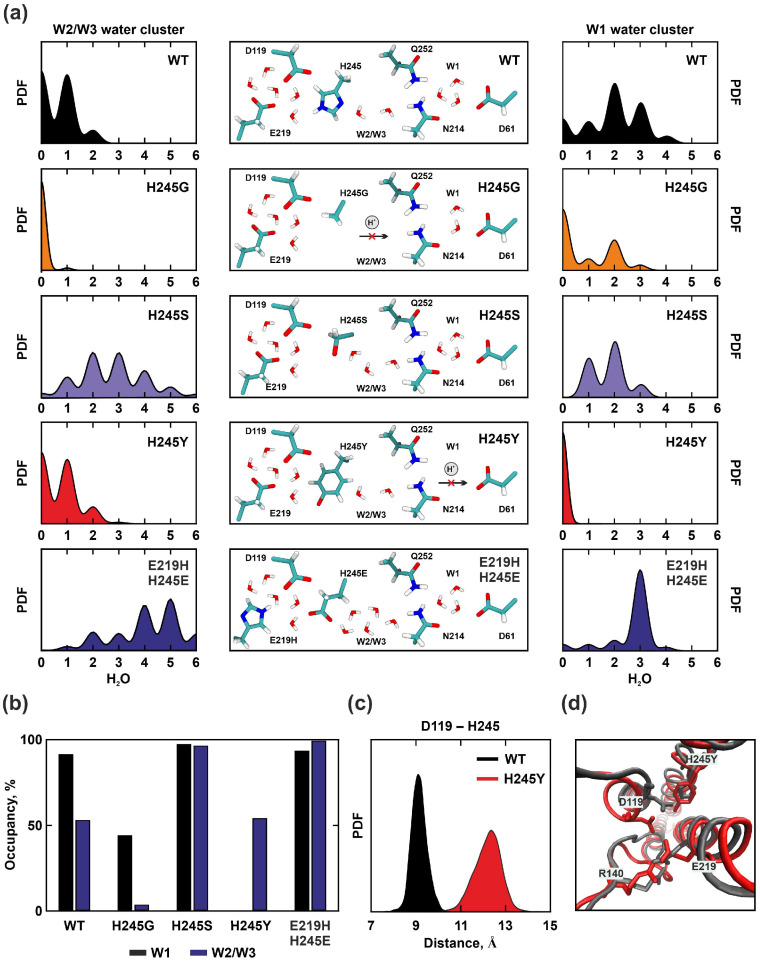
Molecular dynamics simulation results for WT, *a*H245G, *a*H245S, *a*H245Y, and *a*E219H/*a*H245E. (**a**) On the left and right are shown the probability density functions of the water molecules’ number in W1 and W2/W3 clusters localization, reflecting the capacity of the clusters. In the middle are schemes of the possible proton pathway in the bottleneck of the inlet half-channel. In the case of *a*H245G and *a*H245Y substitutions, a break in the transfer chain was observed. (**b**) The water occupancy of clusters is shown in black for W1, blue for W2/W3. (**c**) The probability density function of the distance between the C_α_ atoms *a*D119 and *a*H245 for WT (black) and *a*H245Y (red) indicates an increase in the distance between *a*TMH3 and *a*TMH6 in the gate region. (**d**) Superimposition of the final molecular models obtained at 150 ns simulation for WT (grey) and *a*H245Y (red). The change in the relative position of the *a*E219, *a*D119, *a*R140, and *a*H245 residues side chains forming the gate is demonstrated.

Substitution of the positively charged *a*H245 with a nonpolar glycine having a small side chain led to a decreased hydration of the amino acids *a*N214, *a*Q252, and *c*D61 in the bottleneck (Figure 4a,b), while *a*E219 and *a*D119 were highly hydrated as in the wild type. Up to three water molecules penetrated to *c*D61, therefore the capacity of the W1 cluster was maintained, although the occupancy was reduced (92% for WT and 44% for *a*H245G) (Figure 4b). However, the charge removal resulted in the disappearance of W2 and W3 water molecule clusters, which facilitated the transfer to *a*N214 (Figure 4a,b). Thus, the proton transfer chain was interrupted, as the glycine residue itself, located at position 245, could not participate in the H^+^ transfer.

On the contrary, substitution of the *a*H245 with a polar serine led to increased hydration of almost all conservative amino acids compared to WT (Figure 4a,b). The Ser residue, with a hydroxyl group, could directly participate in the proton transfer to *a*N214. However, the minimum observed distance between them was 5.65 Å which is large for the direct transfer. A chain consisting of an average of two or three water molecules was found between these residues, increasing the capacity of W2 and W3 clusters. Water together with residues *a*E219, *a*D119, and *a*H245S formed a network of hydrogen bonds through which the proton could reach *a*N214; the *a*H245S substitution had no effect on the W1 cluster, and the proton transfer chain was preserved. Thus, solely based on geometric considerations, substitution of the large imidazole ring of histidine with residues having small side chains may not necessarily lead to increased hydration. The polarity of the substituted residue was also found to be a crucial factor in facilitating the water molecules’ penetration into the inlet half-channel and maintaining the proton transport.

Mutation of the *a*H245 to tyrosine featuring a hydroxyl group in the para position of the benzene ring resulted in conformational changes in the *a*-subunit structure. The average RMSD values for this mutant were 2.91 Å. An increase in the distance between *a*TMH3 and *a*TMH6 was observed in the region of *a*D119 and *a*H245Y due to the displacement of *a*TMH3, resulting in a change in the relative positions of the residues forming the gate (Figure 4c,d). This affected the penetration of water molecules into the inlet half-channel towards *c*D61. W2 and W3 water molecules clusters were preserved between *a*H245Y and *a*N214, but water did not infiltrate beyond asparagine to W1 cluster (Figure 4a,b). Thus, the proton transfer chain was interrupted since *c*D61 could not be protonated in the absence of the W1 water molecule cluster.

Experimental substitutions of *a*H245 with small side chain amino acids (Gly, Ser, and Cys) resulted in complete loss of ATP synthase function, as evidenced by the inability of mutated bacteria to grow on a minimal succinate medium. Although some growth for the *a*H245S strain was observed upon addition of 10 to 100 mM sodium acetate [24]. The *a*H245Y substitution led to a complete loss of ATP-coupled proton transport [26]. Therefore, *a*H245 is a functionally important amino acid for the proton transport process. This residue influences the hydration of the inlet half-channel and directly participates in the proton transfer; water molecules or side chains of neighboring polar amino acids cannot replace it.

It is noteworthy that the positions of residues *a*E219 and *a*H245 from *E. coli* are swapped in the mitochondrial ATP synthases of some organisms, such as mammals (*a*H168 and *a*E203) [7] or yeast (*a*H185 and *a*E223) [35]. *a*E219H occupied the same spatial arrangement as in the WT structure, where it was in contact with *a*D119 and *a*H245E almost throughout the simulation. The conserved amino acid residues of the proton transfer chain were more hydrated; there was an increase in the capacity and occupancy of the W1-W3 clusters compared to WT (Figure 4a,b). Thus, the proton trajectory was maintained, as in experimental studies with mutant *E. coli* strains, where the mutual exchange of *a*E219H/*a*H245E positions proved partially functional. ATP-coupled proton transport was observed, although less than that of the WT enzyme [27].

#### 2.3.3. Mutations *a*N214

Another significant amino acid residue for proton transport is *a*N214, whose side chain had stable spatial positions (SP1–SP3) in the wild type enzyme. It was established that *a*N214 in the stable position SP1 was oriented towards *a*H245, while in SP3 to *c*D61 and could protonate it via the W1 water cluster. Thus, the change in the position of the *a*N214 side chain was a necessary condition to ensure proton transport [29].

**Figure 5:**
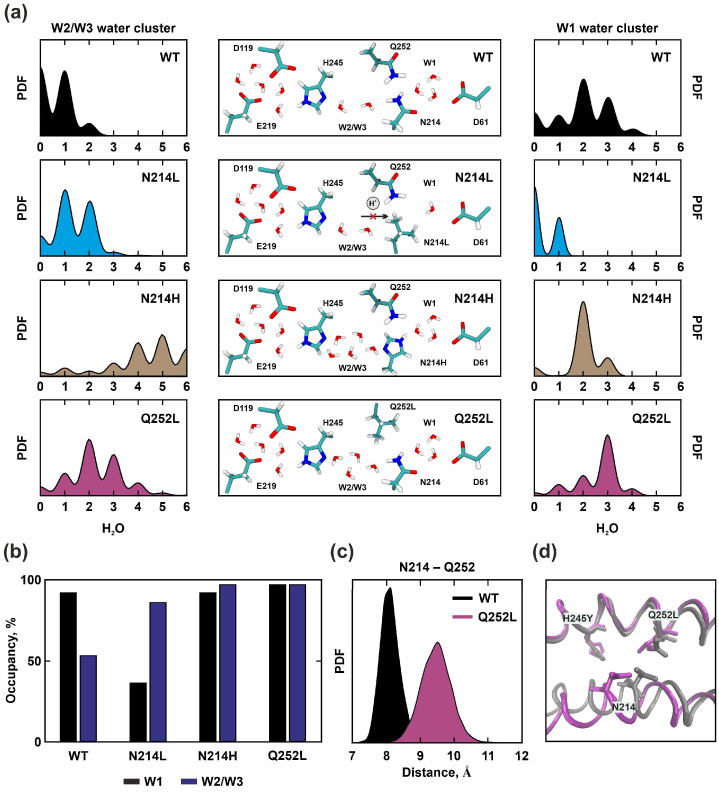
Molecular dynamics simulation results for WT, *a*N214L, *a*N214H, and *a*Q252L. (**a**) On the left and right are shown the probability density functions of the water molecules’ number in W1 and W2/W3 clusters localization, reflecting the capacity of the clusters. In the middle are schemes of the possible proton pathway in the bottleneck of the inlet half-channel. In the case of *a*N214L substitutions, a break in the transfer chain was observed. (**b**) The water occupancy of clusters is shown in black for W1, blue for W2/W3. (**c**) The probability density function of the distance between the C_α_ atoms *a*N214 and *a*Q252 for WT (black) and *a*Q252L (purple) indicates an increase in the distance between *a*TMH5 and *a*TMH6 in the region of *c*D61 residue protonation. (**d**) Superimposition of the final molecular models obtained at 150 ns simulation for WT (grey) and *a*Q252L (purple). The shift of the *a*TMH5 leading to a change in the relative position of the aN214 and aQ252 residues side chains is demonstrated.

When *a*N214 was substituted with nonpolar leucine, the region near *a*E219, *a*D119, and *a*H245 was heavily hydrated and similar to the wild type. Water molecules penetrated into the inlet half-channel up to the *a*N214L, however, the absence of a polar side chain led to difficulties in hydrating *c*D61 and the occupancy of the W1 cluster was reduced (WT–92% and *a*N214L–36%) (Figure 5a,b). Only at the end of the simulation did we observe the penetration of just one water molecule into the W1 cluster region. Thus, the proton transfer chain was disrupted and protonation of *c*D61 was impossible, since the nonpolar side chain of the Leu residue at position 214 could not participate in the H^+^ transfer to W1. Strains with experimental substitutions of *a*N214 with Val, Leu, Gln, or Glu showed a significant decrease in ATP-coupled proton conductance, although the ability of cells to grow in succinate medium was maintained [25].

Interestingly, when *a*N214 was substituted by histidine, the occupancy of the W1 region was close to WT, although the cluster capacity was slightly lower (Figure 5a,b). To clarify, there was no disruption of the H^+^ transfer chain. The proton could reach *a*Q252 or *a*N214H through a chain of water molecules and then protonate *c*D61 via the W1 cluster. However, experimental data on the *a*N214H substitution showed a 95% reduction in enzyme activity and the inability of mutant strains to grow [25].

#### 2.3.4. Mutation *a*Q252

The *a*Q252 residue was located at the end of the inlet half-channel and was in contact with *a*N214, together with which it could protonate *c*D61 via the W1 cluster. However, the *a*Q252 residue was located outside of the “main H^+^ trajectory” and did not play a crucial role in proton translocation, unlike *a*N214.

*a*Q252L substitution resulted in the increased hydration of the inlet half-channel. The capacity and occupancy of the W2/W3 clusters localization were higher (Figure 5a,b); up to four water molecules were observed that coordinated the proton transfer between *a*H245 and *a*N214, whereas in the wild type, there were only a maximum of two. However, the change in cluster W1 was insignificant. Therefore, the *a*Q252L substitution did not disrupt the proton transfer chain but caused conformational changes in the *a*-subunit structure, namely, an increase in the distance between *a*TMH5 and *a*TMH6 in the region of *a*N214 and *a*Q252L, as well as a shift of the *a*TMH5 residues (Figure 5c,d). The average RMSD values for this mutant were the highest of all the concerned mutations and was equal to 2.94 Å.

Experimental studies showed that substitutions of *a*Q252 with uncharged Leu or Val residues reduced the enzyme activity, while substitution with the charged Glu amino acid did not have a significant effect [28]. The results of experiments and MD simulations suggested that *a*Q252 was probably not directly involved in the proton transport process, but its position played an important role in maintaining the stability of the *a*-subunit.

## 3. Discussion

The proton transfer through the membrane F_o_ factor is one of the most crucial steps in the energy transduction process in the ATP synthase. However, the exact proton movement trajectories are poorly understood, in contrast to the *c*-ring rotation and the ATP synthesis reaction itself [36]. Despite the high speed of this process, proton transport is very sensitive to various mutations in the region of half-channels, leading to the protein’s overall dysfunction [37].

In the previous study, we performed MD simulations of the membrane domain of the wild type bacterial ATP synthase embedded in a native lipid bilayer with a PE:CL ratio of 3:1. In the inlet half-channel, the proton transfer chain was identified, consisting of conserved amino acid residues and structural water molecule clusters [29]. This chain served as a basis for the interpretation of MD mutagenesis studies, which provided detailed information about H^+^ transfer at the atomic level. In this investigation, four amino acid residues (*a*E219, *a*H245, *a*N214, and *a*Q252) located in a narrow tract near the key *c*D61 residue of the inlet half-channel were examined. Studying this bottleneck will make it possible to make a more precise assessment of H^+^ translocation parameters, since movement in this region is likely the limiting stage in the proton transport through the half-channels. In order to identify functionally important amino acid residues in the described proton transfer chain, we conducted a mutational analysis using molecular dynamics simulations for 150 ns. By referencing the wild type structure, nine distinct mutations were made in the *a*-subunit using the following substitutions: *a*N214L, *a*N214H, *a*E219G, *a*E219Q, *a*H245G, *a*H245S, *a*H245Y, *a*Q252L, and the revertant *a*E219H/*a*H245E. Our focus was on the impact of these substitutions on the localizations of W1–W3 structural water molecule clusters, as the proton transfer chain was particularly unstable in these regions. It should be noted that cryo-EM structures revealed the densities in the same locations as our clusters. However, the resolution of ~3 Å was insufficient for the precise identification of water [38].

First of all, the effect of all examined mutants on the stability and structure of the *a*-subunit was assessed. The calculated energy landscape of ΔΔG revealed that the protein structure was tolerant to mutations, as substitutions of significant residues did not lead to highly destabilizing effects. The mutated *a*-subunit structures, predicted using AlphaFold alongside the results of MD simulations, also did not show any destructive impact of amino acid substitutions on conformational stability. Meanwhile, *a*H245Y and *a*Q252L substitutions led to changes in the relative position of the *a*TMH3-6 (also reflected in higher RMSD values), influencing the proton transfer chain. We consider that mutations could disrupt the protein activity without altering its stability (or with minor changes), such as by breaking the proton transfer chain or changing the hydration of W1–W3 clusters. Nevertheless, the safe level of stability at which the *a*-subunit of ATP synthase can still function at needs to be established experimentally.

MD mutational analysis of amino acids involved in the proton transfer chain in the inlet half-channel revealed that the residues can functionally effect transport in the following two ways: their participation in direct proton transfer or contribution to half-channel hydration. Thus, in the case of the substitution of the *a*E219 residue with nonpolar glycine or polar glutamine, the possible proton pathway remained largely unchanged compared to the wild type. *a*E219 did not play a crucial role in proton translocation directly since it was located at the boundary of the aqueous cavity and its side chain was heavily hydrated throughout the simulation, i.e., there was an alternative pathway involving a chain of water molecules. However, the change in charge at the bottleneck entrance significantly affected hydration, leading to disruption in the occupancy and the capacity of W1–W3 water clusters. It is worth noting that an occupancy, unlike a cluster capacity, is not a precise assessment parameter, as water molecules can enter the inlet half-channel even during the enzyme assembly stage and remain inside. Nevertheless, a change of this parameter clearly indicates a redistribution of local electrostatic interactions within the half-channel in the case of critical mutations.

The conserved *a*H245 residue played a significant role in the functioning of the proton half-channels, directly participating in proton transfer and influencing hydration. In the case of *a*H245G, the proton transport chain was interrupted because the glycine residue itself could not participate in H^+^ transfer, and no compensatory pathway was found. Water molecules or the side chains of neighboring polar amino acids could not substitute for *a*H245. Conversely, its substitution with the polar serine increased hydration of the inlet half-channel, leading to the emergence of an alternative pathway via the chain of water molecules. In the case of the *a*H245Y mutation the hydrophobic benzene ring with a hydroxyl group caused conformational changes in the structure of the *a*-subunit and did not facilitate the penetration of water molecules into the bottleneck. This led to disruption of hydration and, as a consequence, to the disappearance of the W1 water cluster.

In the case of *a*E219H/*a*H245E exchange, corresponding to mitochondrial ATP synthases, the proton trajectory remained unchanged. This interchangeability of nearby amino acids allows different organisms to use unique sets of polar residues to form their proton half-channels, and their variability suggests an incredibly flexible mechanism for proton transfer in a highly efficient macromolecular machine.

The *a*N214L substitution located in the middle of the narrow tract resulted in the impossibility of proton transport through the inlet half-channel, associated with the inability of the Leu residue to participate in proton transfer. The *a*N214H substitution did not affect the occupancy of the W1, but decreased its capacity, and the proton transfer chain remained intact. Interestingly, experimental studies of the *a*N214H mutant strains of *E. coli* demonstrated a 95% decrease in enzyme activity. It is possible that the observed protein dysfunction was not caused by the blocking of the proton transport. *a*N214 is located near the highly conserved *a*R210 residue, which is responsible for the rotor rotation associated with proton transport [12]. The *a*N214H substitution led to a strong positive charge in the protonation region of *c*D61, which in turn can cause disruption of the *c*-ring rotation. The development of genetic approaches, as well as improving methods for recording and analyzing proton transport across membranes, will help clarify the discrepancies between the results of MD simulations and experimental data with mutant strains of *E. coli*. Additional studies on the rotation of the *c*-ring will also allow for a more precise assessment of which the process (H^+^ transport or rotation) is influenced by mutations in the *a*-subunit.

The *a*Q252 residue appeared to not be critical for proton transport as there was an alternative path through water molecules and other amino acids upon its substitution; the proton transfer chain was not disrupted. However, the observed conformational changes indicated the importance of this residue in maintaining of the stability of the *a*-subunit. Generally, an increased hydration allows for the enhancement of the rate of proton movement during enzyme functioning and the facilitation of transport under conditions of critical mutations. However, for the efficient functioning of these pathways, the presence of strictly oriented chains of water molecules might be necessary. Otherwise, increased hydration can lead to random proton wandering in the half-channel or deviation from the main trajectory of its movement towards residues that may act as proton traps.

As proposed earlier, the proton translocation path to the center of the membrane appeared to be a complicated arrangement of water-filled cavities adjacent to narrow nonlinear tracts lined with polar amino acids [16]. The requirement in obligatory structure water clusters (W1–W3) and definite amino acid residues spatial positions (SP) predicates the system to be very sensitive to any interventions. However, the precise MD analysis of the most vulnerable part, the bottleneck, showed the substantial structural stability of the F_o_F_1_-ATP synthase inlet half-channel to the selected mutations in *a*-subunit. The *a*H245 and *a*N214 residues appeared to be critical for the proton transport process; they directly participated in H^+^ transfer and their substitutions with nonpolar amino acids disrupted the assumed way of protons transduction, as water molecules or side chains of neighboring polar amino acids could not substitute them. At the same time, the *a*E219 and *a*Q252 residues seemed not to play a decisive role in proton translocation since there was an alternative pathway involving a chain of water molecules when they were replaced. The existence of the hydration self-regulative mechanism inside the enzyme such as the “gate” (including *a*E219, *a*D119, and *a*H245) located at the aqueous cavity exit to the bottleneck raises new questions about the direct and indirect role of the exact amino acids. All the substitutions concerned in the paper had a valuable effect on hydration, leading to significant variations in the occupancy and the capacity of the W1–W3 water clusters, up to their complete disappearance, and consequently, to the disruption of the proton transfer chain. However, the unobvious detected impact of the selected residues substitution, including the change in their charge and/or side chain size, on the continuity of the proton translocating pathway, definitely inspires more profound experiments in mutational analysis.

Based on the MD trajectories, we described the behavior of significant amino acids and water molecules in the inlet half-channel involved in the formation of the possible proton transfer chain and located at a distance of up to 3 Å (the approximate length of a hydrogen bond). However, the actual H^+^ transfer was not simulated in this study. Molecular dynamics simulations proved to be useful in predicting how the proton transfer chain in the inlet half-channel of the bacterial ATP synthase will respond to changes such as mutations. Further research on deciphering the molecular mechanism of proton transfer considering various factors will uncover the aspects of the cellular energy supply in health and disease.

## 4. Materials and Methods

### 4.1. System Preparation

Based on the Cryo-EM crystal structure of *E. coli* F_o_F_1_-ATP synthase (PDB ID: 6VWK), we constructed an atomistic molecular model of the F_o_-ATP synthase wild type membrane factor, including the proton-conducting *a*-subunit and a ring of ten *c*-subunits, as well as truncated peripheral stalk *b*_2_-subunits (residues 4 to 35). The most probable protonation states at pH = 6.5 of all titratable residues were determined using the H++ web server, where the protein interior and the solvent were treated as continua with dielectric constants ε_int_ = 2 and ε_ext_ = 80, respectively [39]. Notably, this resulted in protonation of the key *c*Asp61 of all *c*-subunits except aspartate facing the lumen of the inlet half-channel.

The protein was embedded in a lipid bilayer oriented perpendicular to the z-axis using the CHARMM-GUI Membrane Builder program [40] and consisting of 206 DYPE (1,2-dipalmitoleic-phosphatidylethanolamine), 96 DPPE (1,2-dipalmitoyl-phosphatidylethanolamine), 96 PMPE (1-palmytoil-2-cis-9,10-methylenehexadecanoyl-phosphatidylethanolamine), 108 PMCL^2−^ (1,2-dipalmitoyl-1′-palmitoil-2′-cis-9,10-methylenehexadecanoyl-cardiolipin) and 36 TYCL^2−^ (1,2-1′,2′-tetrahexadecenoyl-cardiolipin) molecules (73% PE and 27% CL), reflecting the composition of the inner bacterial membrane [41]. One DYPE molecule per leaflet was manually inserted into the *c*-ring to seal its central channel. The system was solvated with 36785 TIP3P water molecules in a rectangular box of size 150 Å × 150 Å × 90 Å and the number of K^+^ and Cl^−^ ions was adjusted to maintain a salt concentration of 150 mM and to neutralize the net charge of the system. The details of the molecular model of the wild type enzyme have been described elsewhere [29]. 

To investigate the role of certain amino acids in the proton transport process, a mutational analysis was performed. The mutations included changes in the residue’s polarity, their position in the polypeptide chain or the length of the side chain. Additionally, the selection of mutants was guided by experimental data, since most functionally significant amino acids have been identified and studied using *E. coli* mutant strains [22,23,24,25,26,27,28]. Based on the molecular model of the wild type membrane-embedded F_o_ factor, the mutated systems were constructed via the following substitutions: *a*E219G, *a*E219Q, *a*H245G, *a*H245S, *a*H245Y, *a*N214L, *a*N214H, the revertant *a*E219H/*a*H245E, and *a*Q252L using the Mutator plugin in VMD [42]. In systems where residue substitutions created a nonzero net charge, the number of ions in the solvent was adjusted to neutralize the system.

### 4.2. Prediction of a-Subunit Destabilization

To evaluate the impact of mutations on the stability and structure of the ATP synthase *a*-subunit, we calculated the change in the Gibbs free energy of protein folding (ΔΔG). The change in ΔΔG between wild type and mutant proteins was computed using FoldX version 5.0 [31] and Eris version 1.0 with a flexible backbone protocol [32]. These tools directly manipulated the three-dimensional structure of a protein and used empirical force fields (obtained from protein engineering experiments) with a fast conformation-sampling algorithm in an atomic framework of proteins. This made it possible to estimate the free energy of folding of wild type (ΔG_WT_) and mutant (ΔG_Mut_) proteins. The difference in free energy is given by ΔΔG = ΔG_Mut_ − ΔG_WT_. Additionally, the 3D structures of mutants were predicted using AlphaFold v2.3.2, although it has not been validated for predicting the effects of mutations [34].

### 4.3. Molecular Dynamics Simulation Parameters

All molecular dynamics (MD) simulations were performed with NAMD software version 2.14 [43] and CHARMM36 force field [44,45]. The simulations were conducted in the isothermal-isobaric (NPT) ensemble with periodic boundary conditions. The constant temperature was maintained at 310 K using a Langevin thermostat and the pressure was kept at 1 bar through the application of the anisotropic Langevin piston barostat. Long-range electrostatic interactions were evaluated using the particle mesh Ewald (PME) method, and the cutoff between short-range and long-range was set to 12 Å. Van der Waals interactions were evaluated with a smooth cutoff at 12 Å and a switching distance of 10 Å. The equations of motion were integrated with a time step of 2 fs, and the coordinates were recorded every 2 ps. After the energy minimization for the gradual relaxation of the membrane and protein, each mutant model system was equilibrated in the NPT ensemble for 15 ns with the protein atoms harmonically restrained by a force constant of 2 kcal/mol/Å^2^. This was followed by a production run, where the MD simulation was carried out without restraints for 150 ns each. The VMD program [42] and customized Tcl scripts were utilized to perform the visualization and analysis of the obtained trajectories.

## Figures and Tables

**Table 1:** The calculated parameters reflecting the structural stability of *a*-subunit for all systems, obtained using MD modeling or AlphaFold.

Mutations	RMSD, Å	RG, Å	SASA, 10^4^ Å^2^	No. of Hbond	RMSD (AlphaFold), Å
WT	2.41	22.24	1.25	59	1.51
*a*E219G	2.67	22.15	1.26	61	1.51
*a*E219Q	2.60	22.46	1.30	61	1.49
*a*H245G	2.36	22.31	1.26	59	1.73
*a*H245S	2.45	22.40	1.28	59	1.61
*a*H245Y	2.91	22.44	1.37	61	1.89
*a*E219H/*a*H245E	2.51	22.20	1.29	57	1.67
*a*N214L	2.64	22.34	1.30	61	1.48
*a*N214H	2.59	22.47	1.32	60	1.43
*a*Q252L	2.94	22.37	1.32	58	1.48

## Data Availability

All material that could be shared is in this paper.
